# Reversibility of Spermatogonial Stem Cell Injury After Single Acute Scrotal Hyperthermia in Mouse and Rat Models: A Systematic Review

**DOI:** 10.1002/rmb2.70055

**Published:** 2026-05-11

**Authors:** Rashid A. Aldahhan

**Affiliations:** ^1^ Department of Anatomy, College of Medicine Imam Abdulrahman Bin Faisal University Dammam Saudi Arabia

**Keywords:** germ cells, hot temperature, Rodentia, spermatogenesis, spermatogonia, testis

## Abstract

**Background:**

Acute scrotal hyperthermia is a recognized spermatogenesis stressor, but recovery of spermatogonial stem cells after a single mild heat exposure remains unclear, particularly over cycle‐relevant timeframes. This systematic review evaluates the in vivo local scrotal hyperthermia across at least one full spermatogenic cycle.

**Methods:**

A PICO‐driven search identified 18 mouse and rat studies using a single scrotal heating exposure. Outcomes were synthesized thematically across structural, cellular, endocrine/apoptotic, and sperm domains.

**Results:**

Injury patterns were graded and temperature‐ and species‐dependent, with more consistent abnormalities at 43°C and milder effects in rats than in mice. Structural endpoints often remained abnormal at the longest follow‐up, and architectural repair did not reliably achieve functional recovery. Mouse stereology at 43°C indicated long‐term SSC and germ‐cell depletion, with Leydig‐cell and partial Sertoli‐cell losses; rat stereology was sparse and absent at 42°C. Testosterone responses were heterogeneous, whereas tubular apoptosis was more consistently elevated in mice at 43°C. Sperm concentration/count declined where assessed, with more frequent impairments of motility and other quality indices, including DNA integrity, at 43°C.

**Conclusion:**

Recovery was often incomplete and endpoint‐specific, with impairment more consistently detected at 43°C, particularly in mice. Future studies require standardized thermal dosing, transparent bias‐mitigation, and SSC/niche‐resolved functional assays.

## 1 Introduction

Spermatogenesis, the intricate process of mammalian male germ cell production, requires a maintenance temperature that is 2°C–7°C below normal body temperature to sustain the function of both germ and somatic cells. Spermatogonial stem cells (SSCs) are the diploid stem cell precursors of all germinal epithelial cells [1]. In this review, the abbreviation “SSC” is used broadly to refer to spermatogonia in general, unless otherwise specified. In rodents, A_single_ (A_s_) SSCs that reside in the basal compartment of the seminiferous tubule are derived from gonocytes, which in turn originate from primordial germ cells [2]. These SSCs have a dual capability, as they serve in both SSC pool maintenance and germline lineage commitment by differentiating into A_paired_ (A_pr_) SSCs, which further divide to give rise to A_aligned_ (A_al_) SSCs [1, 3]. These SSC types, termed undifferentiated spermatogonia, have the capacity to differentiate into A_1_–A_4_, intermediate and B spermatogonia, which then enter meiotic divisions to produce haploid spermatids [4, 5]. SSC differentiation is a highly complex process that demands tight regulation; therefore, disruption can lead to infertility [1].

An extensive body of literature highlights the adverse impacts of transient scrotal/testicular hyperthermia, such as those occurring during febrile illness, short sauna or hot‐bath exposures, occupational heat exposure, varicocele or experimentally induced cryptorchidism. This heat exposure can perturb the functioning of seminiferous epithelial cells through processes involving oxidative stress, mitochondrial dysfunction, heat‐shock responses and disruption of the blood–testis barrier [6, 7, 8]. However, an important point to note is that the biological consequences of a single acute heat exposure differ from those of repeated or chronic episodes. This is because cumulative injury, adaptive responses and prolonged niche perturbation can obscure the interpretation of true reversibility after an isolated insult. This distinction is also relevant to human exposure scenarios involving brief transient heating, such as those occurring during hot bathing or similar lifestyle‐related thermal exposures in which the longer‐term implications for male reproductive function remain incompletely defined.

Most studies conducted over the past decades have focused on the heat response of meiotic and post‐meiotic germ cells, and the results have revealed a consistent hierarchy for the vulnerability of germ cells. The most heat‐sensitive cells are the pachytene spermatocytes and round spermatids, which undergo rapid apoptosis after hyperthermia [8]. By contrast, thermal effects on SSCs have received less attention, and divergent observations have been reported. Early studies suggested that SSCs are the germ cells least susceptible to thermal damage following mild testicular heating [9, 10]. However, extended observations of heat impacts on rat testes over two or more spermatogenic cycles have indicated a negative influence of heat on SSC differentiation and proliferation, as evidenced by a persistent decrease in testis weight for up to 6 months after heat exposure [11, 12, 13]. Consistent with these findings during extended follow‐ups, McLean et al. [14] reported a marked enrichment of transplantable murine SSC activity 15 days after testicular heating (43°C, 15 min), suggesting preferential survival of SSCs relative to differentiating germ cells. Conversely, Paul et al. [15] demonstrated that a single episode of scrotal thermal stress (42°C for 30 min) increased cleaved caspase‐3 immunoreactivity near the basement membrane and among differentiating germ cells, with minimal effect at 40°C. These data suggest that even brief scrotal hyperthermia can drive robust and temperature‐based caspase‐3–mediated apoptosis in SSCs, thereby challenging the notion that SSC populations are less vulnerable to heat stress than other germ cells.

In vitro studies provide additional mechanistic context, but their short observation windows and lack of an intact testicular niche limit direct inference regarding long‐term in vivo recovery. A single transient hyperthermia exposure has been shown to curtail proliferation in a CD1 mouse–derived SSC line, disrupt transcriptional and cell‐cycle programmes within hours, and suppress differentiation under repeated short‐term exposure [16, 17]. However, because these observations are confined to hours or days after heat exposure, they do not determine whether these types of perturbations are reversible if followed across a full in vivo spermatogenic cycle.

The available literature clearly indicates that chronic heat exposure, provided either in vivo or in vitro, damages the function and structure of germ cells, including SSCs. Evidence from mice, rats and humans supports some degree of heat‐related functional impairment arising from epigenetic deregulation, reduced DNA synthesis and disrupted differentiation or regeneration [18, 19, 20]. In developmental models, such as genetically induced cryptorchid murine testes or flutamide‐induced perinatal cryptorchid rat testes, heat exposure has been associated with impaired SSC establishment, altered self‐renewal/differentiation balance and subsequently compromised spermatogenesis [20, 21]. These models are informative, but they address a biological question that is distinct from whether a single acute heat exposure causes reversible or durable SSC‐related injury. Therefore, the discrepant reports of SSC resistance versus vulnerability, together with the short in vitro observation windows used, emphasize the need for an in vivo assessment of SSC fate and recovery across at least one full spermatogenic cycle.

To date, most experimental studies have employed rodent models, particularly rats and mice. Accordingly, the aim of this paper is to isolate the recovery trajectory after a discrete thermal insult by providing a systematic review of rodent studies that have investigated the in vivo effects of acute mild hyperthermia on SSCs over one or more spermatogenic cycles (49 and 35 days in rats and mice, respectively). The overall purpose is to evaluate and synthesize the available evidence regarding the reversibility of acute heat‐induced SSC damage in these rodents. By focusing specifically on a single acute exposure and cycle‐aligned follow‐up, this review seeks to distinguish transient post‐heat perturbation from the more durable impairment of SSC‐driven regeneration. The review particularly addresses the nature and severity of acute heat‐induced SSC damage, the time course and characteristics of recovery, and the influence of key exposure‐related factors, such as temperature and duration.

## 2 Methods

### 2.1 Protocol and Reporting Framework

A detailed protocol covering objectives, eligibility criteria, search strategy and analysis plan was finalized before database searches were conducted and was not prospectively registered. The review was carried out exactly as prespecified, with no methodological deviations and full adherence to the Preferred Reporting Items for Systematic Reviews and Meta‐Analyses (PRISMA) 2020 guidelines [22].

All review stages, including screening, eligibility assessment, data extraction, and risk of bias appraisal, were conducted by a single reviewer. To minimize error, each stage underwent a blinded second‐pass verification (first‐pass decisions hidden and rows re‐randomized) at least 7 days later, with any records flagged as uncertain during screening then undergoing rechecking against the full texts. All extracted data were verified against source tables and figures, and risk‐of‐bias ratings were re‐evaluated domain by domain. Any discrepancies were resolved by returning to each source publication, and amendments were documented to maintain a transparent audit trail.

### 2.2 Search Strategy

This systematic review was planned and reported in accordance with the PRISMA guidelines [22]. The systematic search was performed from database inception in PubMed, Scopus, Web of Science, and Embase during May 6–13, 2025 (database‐specific search dates are provided in Table S1). These databases collectively provide a broad coverage of biomedical research relevant to reproductive biology, experimental animal studies, and stem‐cell biology. Searches were limited to English‐language records published from January 2005 to May 2025 in each database. Despite this language filter, occasional non‐English citations were encountered; these were screened and evaluated against the eligibility criteria (see below).

A multi‐concept strategy was constructed to review three main elements: (1) spermatogonial stem cells and related germline/testicular cell terms; (2) acute heat or hyperthermic exposure; and (3) rodent models. Controlled vocabulary (e.g., MeSH, Emtree) and free‐text keywords were combined using synonyms, truncation, and Boolean operators. The core title/abstract text‐word string was as follows:

(“spermatogonial stem cells” OR SSC OR “germline stem cells” OR “testicular stem cells” OR spermatogonia OR “spermatogonial progenitor cells” OR “spermatogenic stem cells” OR “male germline stem cells” OR “testicular germ cells” OR “germinal epithelium” OR “spermatogonial niche”) AND (“acute heat” OR “acute heat exposure” OR “heat stress” OR “thermal stress” OR hyperthermia OR “testicular hyperthermia” OR “scrotal hyperthermia” OR “heat shock” OR “heat induced damage” OR “thermal injury” OR “scrotal temperature elevation” OR “testicular temperature elevation” OR “acute thermal exposure”) AND (rodent* OR rat OR rats OR mouse OR mice OR murine OR “ *Rattus norvegicus* ” OR “ *Mus musculus* ”).

Database‐specific syntax (field tags, subject‐heading explosions) was adapted as required. Bibliographies of the screened and included studies were examined to identify additional eligible reports not captured by the electronic searches.

### 2.3 Eligibility Criteria

A priori inclusion and exclusion criteria were specified to ensure consistent study selection. Eligible reports met all of the following: (1) peer reviewed, original, full text article; (2) published January 2005–May 2025; (3) English language; (4) randomized or nonrandomised in vivo study of a rodent model (rat or mouse); (5) acute, single, localized heat exposure to the testes/scrotum; (6) an analyzable comparator permitting inference on post‐heat recovery—nonheated/sham controls, or heat only versus heat + co‐exposure/intervention arms (e.g., magnetic field) [23], or pre‐exposure baseline; and (7) report of ≥ 1 pre‐specified outcome relevant to reversibility of SSC damage or downstream spermatogenesis recovery. Outcomes were grouped prospectively into four thematic categories applied throughout the review, where (1) Structural integrity (e.g., histology, seminiferous tubule diameter, testis weight/index) and (2) Stereology (SSC, germ lineage, and somatic cell counts) together constituted the Primary SSC/germinal recovery endpoint, while (3) Endocrine/apoptosis (testosterone, %TUNEL/ssDNA positive tubules) and (4) Sperm parameters (count/concentration, motility, morphology, viability, TUNEL positive sperm) together constituted Secondary downstream spermatogenesis endpoints. Studies reporting at least one outcome in any theme were eligible; reporting across all themes was not required.

Exclusion criteria encompassed the following: non‐original publications (reviews, commentaries, editorials, letters, conference abstracts without full data), non‐rodent or human studies, and irrelevant heat protocols (chronic, repeated or ambient/environmental heat lacking an acute component). Studies in which all animals received the same acute heat exposure but differed only by a post‐heat co‐exposure/intervention remained eligible. Additional exclusions were out‐of‐scope outcomes (no SSC/spermatogenesis recovery, no reproductive endpoints); studies consisting only of in vitro or *in silico* investigations; and reports without an accessible English full text.

### 2.4 Study Selection

Search results from all databases were imported into EndNote and deduplicated (automatic match + manual review). The deduplicated set was then screened at the title/abstract level against the a priori eligibility criteria. Records clearly failing any criterion (non‐rodent, nonoriginal, in vitro‐ only, chronic heat, etc.) were excluded at this stage. Multiple publications arising from the same experimental cohort (e.g., conference posters, secondary analyses) were screened for overlap. When duplicate data were identified, the most complete peer‐reviewed article was retained, and secondary sources were excluded to prevent double‐counting (recorded as “duplicate reports” in the screening log [Table S2]). One non‐English report was identified, despite the English language search filter being excluded at this stage because no English full text was available. When eligibility could not be determined from the title/abstract (particularly where the heat protocol or outcome scope was unclear), the record was retained for full‐text assessment to minimize erroneous exclusion.

Full texts were retrieved for all records that passed screening or were uncertain. Each full text was evaluated against all inclusion criteria; reasons for exclusion were recorded in a study selection log and are summarized in Table S3. Three reports progressed to full text review because their abstracts did not specify the heat protocol or outcomes; all three were subsequently excluded when the full text confirmed that the required acute heat exposure and/or SSC‐related outcomes were absent.

**FIGURE 1:**
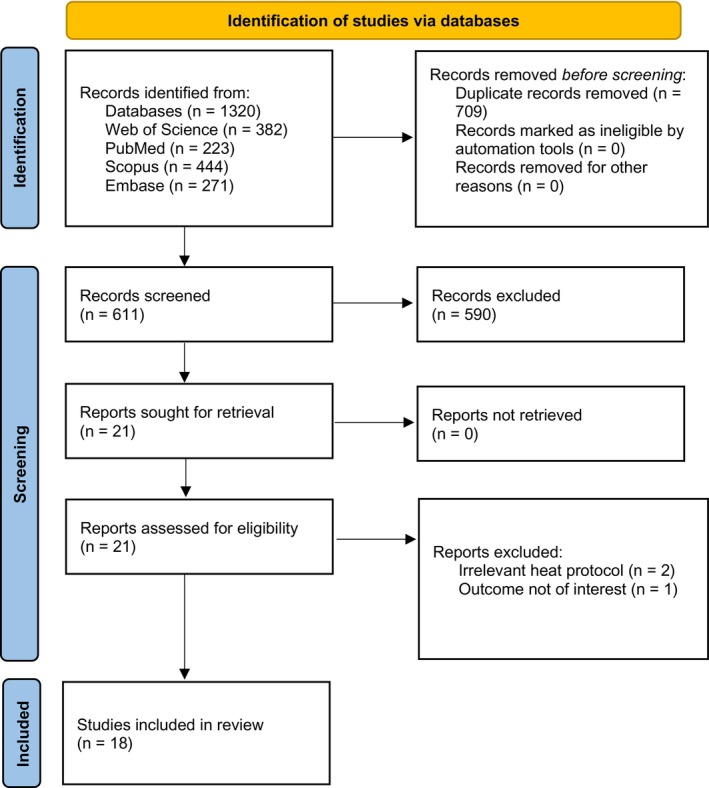
PRISMA flow diagram summarizing the identification and selection of included studies. *Source*: Page et al. [22]. This work is licensed under CC BY 4.0.

After all exclusions, 18 studies met the inclusion criteria and were incorporated into the qualitative synthesis. The study selection process is depicted in the PRISMA flow diagram (Figure 1).

### 2.5 Data Collection

Data were extracted into a structured spreadsheet using predefined fields. Captured elements included the following: bibliographic descriptors (first author and year); experimental characteristics (rodent species, strain, age/weight, housing temperature when reported); heat exposure protocol (acute, single, local scrotal/testicular heating only—peak temperature, duration, heating protocol); postexposure recovery timepoints; group sizes (*n/group*); and reported outcomes mapped to the four prespecified thematic categories (Structural integrity; Stereology; Endocrine/apoptosis; Sperm parameters; see Eligibility). For each outcome, the extracted data comprised measurement type, units, timepoint(s) and statistical significance versus comparator, and direction of effect (↑increase/↓decrease/↔ no difference) or, where quantitative data were unavailable, a qualitative evaluation (e.g., histological integrity). Long‐term outcomes were flagged when assessments occurred at or beyond one full spermatogenic cycle for the species (35 days for mice; 49 days for rats) to aid interpretation of recovery.

After initial extraction, all fields were rechecked in a second blinded pass at least 7 days later (washout) to reduce transcription error and subjective drift; discrepancies were resolved by returning to the source publication. In this solo‐authored review, this delayed second‐pass check was used as an internal quality‐control step to minimize avoidable extraction error, recognizing that independent second‐reviewer verification, which is recommended in streamlined rapid‐review guidance, was not feasible [24]. This safeguard was prioritized because the associated error rate is higher for single data extraction than for duplicate extraction [25]. Single‐authored systematic reviews using PRISMA‐referenced reporting have been published, supporting the feasibility of a solo workflow when transparency is maintained [26]. The second‐pass check was also used to harmonize terminology across studies (e.g., reconciling seminiferous tubule diameter vs. lumen diameter; mapping germ‐cell stage nomenclature to standard categories). Derived variables created for synthesis included the temperature band (≥ 43°C vs. < 43°C), follow‐up duration band (< spermatogenic cycle vs. ≥ cycle), and sentinel outcome flag (outcomes reported in ≥ 3 studies overall). When numerical data were insufficient for effect size calculation, direction‐only and qualitative descriptors were retained.

The study characteristics for all included reports (species, heat protocol, recovery intervals, key outcomes, and RoB) are summarized in Table 1.

**TABLE 1:** Summary of the study characteristics for all included reports (species, heat protocol, recovery intervals, key outcomes, and RoB).

Study ID	Country	Species/strain	Heat exposure (°C × min)	Recovery time‐points (days)	Key long‐term outcomes (≥ 35/49 days)	*n* per group	Overall RoB	Notes
Lee et al. 2020	USA	Mouse/C57BL/6	42°C × 15 min	0, 14, 28, 42	Testis index, % abnormal tubules, histology	3–6	High	No sperm, hormone or ROS data at 42 days.
Aldahhan et al. 2019	Australia	Rat/Sprague–Dawley	43°C × 15 min	7, 14, 28, 56, 84, 98	Histology, Testis weight, no. TUNEL +ve, Serum inhibin, Hspa1a and Hspa1b mRNA	7	High	TUNEL data measured but not shown.
Chihara et al. 2014	Japan	Mouse/B6, MRL, B6.MRLc1 (A/J, AKR/N, BALB/c, C3H/He, WBB6F1‐þ/þ, WBB6F1‐W/Wv, ddY, and ICR mice)	43°C × 20 min	1, 2, 3, 10, 20, 40, 60, 110	Macroscopic exam, % von Kossae positive tubules, energy‐dispersive X‐ray microanalysis	NR (3–9)	Unclear	Sample size per time‐point not stated
Chihara et al. 2015	Japan	Mouse/C57BL/6 B6.MRLc1, B6.MRLc11, B6.MRLc1c11 MRL/MpJ	43°C × 20 min	1, 2, 3, 10, 20, 60	Testis weight, IHC: (numbers of STRA8 and DMC1‐positive cells), SC number (SOX9 +ve), % von Kossae positive tubules, apoptosis: total number of ssDNA‐ positive cells/ST‐CS	NR (≥ 3)	High	Exact sample size per time‐point not stated. Unit‐of‐analysis error (tubule counts treated as independent). Possible selective histology reporting (heat images absent). Genotype confounding (multiple strains with different baseline testis size).
Dong et al. 2016	China	Mouse/C57BL/6	43°C × 20 min	1, 14, 35, 70	Pregnancy rate, litter size, sperm count, motility & morphology, serum cholesterol, triglycerides, glucose, and sex hormones	10	High	Unclear for the developmental‐age arm (P5–P35) where only “3 ≤ *n* ≤ 7” ranges are given. Hundreds of tubules were counted per testis, but statistics were expressed as *n* = mice (possible pseudoreplication) for apoptosis and lipid‐droplet assays. The additional Txlna‐knockdown arm introduces a recovery‐modifying genetic intervention; combining it with heat‐only groups would confound attribution of effects to hyperthermia. No evidence of selective non‐reporting for long‐term outcomes (all stated endpoints appear).
Hasani et al. 2020	Iran	Mouse/NMRI	43°C × 30 min	70	Sperm count, motility, morphology & vaibility sperm chromatin dispersion (SCD) index, TUNEL DCF absorption, GPX activity U/mL, number of SSC, PSyt & rST, ST lumen diameters, testis weight, histology, serum T, GSH concentration, LC & SC number, IL1‐α, IL6 & TNF‐α mRNA	6	Unclear	Laser co‐intervention excluded.
Ilkhani et al. 2020	Iran	Mouse/NMRI	43°C × 30 min	35	Sperm count, motility, morphology & vaibility testis and interstitial tissue volume & ST volume, testicular cells apoptosis (TUNEL +ve cells%), number of SSC, PSyt & rST, c‐kit, STRA8, and PCNA mRNA, LC & SC number, serum T, cross‐correlation and spatial arrangement and of testis cells	8	Unclear	The Method only mentioned sperm counting method while ignoring how other parameters were measured. Sperm assay method was not reported for viability/motility/morphology
Kheradmand et al. 2011	Iran	Rat/Wistar	43°C × 15 min	10, 30, 60	Histology, diameter of ST and Sg & SCs nuclei, epithelial height, miotic index, spermatogenesis rate % & presence of Syt%, %volume density of main testicular components: tubular lumen, germinal epithelium & interstitial space, no immunoractivity Bcl2 IHC	5	High	Selective‐reporting bias: organ‐mass results & Bcl‐2 60 days IHC mentioned but omitted; (selective reporting for timepoint [60 days data were not shown]).
Kheradmand et al. 2012	Iran	Rat/Wistar	43°C × 15 min	30, 60	IHC ‐Bax expressions, IHC ‐PCNA expressions [Sg & Syt containing PCNA (%)], IHC‐Bcl‐2 expressions (30, 60), Sg & Syt immunoreactivity	5	Unclear	Results reporting below average.
Khosravi et al. 2020	Iran	Mouse/NMRI	43°C × 30 min	70	Sperm count, motility & vaibility, SCD index (SCD%), TUNEL(Apoptotic cells%), testicular volume, ST length density, number of SSC, PSyt & rST, ST lumen diameters, testis weight, histology, serum T, LC & SC number, IL1‐α, IL6 & TNF‐α mRNA	6	Unclear	—
Liu et al. 2012	China	Mouse/C57BL/6J	42°C × 30 min	2, 7, 14, 42	Histological integrity	4	Unclear	Strain: Wild‐type (background not specified; likely C57BL/6J based on MMRRC source).
Melo et al. 2022	Brazil	Rat/Wistar	42°C × 12 min	7, 15, 30, 60	Fractal dimension, lacunarity, multifractality, quantitative morphometry	7	Unclear	—
Mohajeri and Kaffashi 2015	Iran	Mouse/Balb/C	43°C × 15 min	50	Lipid peroxidation [(MDA) oxidative stress], activities of antioxidant enzymes (SOD, CAT & GPx), STs diameter and spermatogenesis, histopathological observations, serum T	10	Unclear	Histology was scored “in a blind fashion” by an expert pathologist. No statement on blinding during heat procedure or sampling. Ethical approval was not reported
Moradpour et al. 2018	Iran	Rat/Wistar	43°C × 15* min	15, 30, 60	Acute response only reported	6	High	Heat duration was not stated for the 43°C bath; assumed 15 min because the sham was 23°C × 15 min. Authors autopsied at 60 days but present no TDI/RI/SI or histology for the 43°C group; they only noted “restoration progress.” No extractable data. Methods: heat‐saline rats autopsied at 15–30–60 days Housing randomization and funding source were not described.
Pérez‐Crespo et al. 2008	Spain	Mouse/CD1	42°C × 30 min	7, 14, 21, 28, 60	Sperm concentration, viability, motility, progressive motility, TUNEL assay (% TUNEL +ve spermatozoa)	5	Unclear	No description of how animals were allocated Methods specify *n* = 5 males per treatment at every sperm‐analysis time‐point and *n* = 10 males per treatment for mating tests; all figures/tables use the planned numbers. No deaths/exclusions reported Assay validation mentioned (data not shown) Results showed TDI/RI/SI only for 30 days; histology images only for the 30 days heat and 60 days treated groups → selective outcome reporting.
Queiroz et al. 2013	Brazil	Rat/Wistar	43°C × 15 min	3, 7, 15, 30, 60	Histological integrity, GCs nuclear fragmentation, serum T, testis weight (statistically insginifcant likely d/t low sample size), ST volume, daily sperm production per testis or gram of testis, SC index and rST per ST cross section, SC population	3	High	Sample size is an issue (*n* = 3/timepoint/group) Paper states “72 rats in total” (18/group). Text also mentions “*n* = 6 per group per day,” which corresponds to six testes (= 3 rats) sampled at each of the 5 time‐points. Selective outcome reporting: the Methods specify that four germ‐cell subtypes were counted (pre‐leptotene, pachytene, round spermatids, Sertoli) whereas in the Results only round spermatids and Sertoli‐related indices are shown (counts for the two spermatocyte classes are omitted). Housing randomization, funding and conflict of interest were not reported.
Tenorio et al. 2013	Brazil	Rat/Wistar	43°C × 12 min	15, 30, 60	Seminiferous epithelium volume lumen volume ST total volume Gonadosomatic index Tubular area & perimeter Histology Testis weight (inferred data) Plasma T	6	Unclear	Because the study included no unheated control, it does not inform the magnitude of heat‐induced damage itself. It only shows that magnetic‐field exposure impairs recovery after an identical heat insult. “The absence of a negative control does not raise attrition or sequence bias, but it limits external validity.”
Zhang et al. 2020	China	Mouse/C57BL/6J	42°C × 20 min	6 h, 12 h, 1, 2, 7, 14, 21, 35	Histological integrity, testis index, % abnormal tubules	5	Unclear	Exact attrition was not reported. No markers for apoptosis, oxidative stress, autophagy, SSCs or BTB markers were examined for long‐term heat effect.

### 2.6 RoB Assessment

All ten Systematic Review Centre for Laboratory Animal Experimentation (SYRCLE) signaling questions were extracted and then aggregated into four reporting‐level domains: (1) Selection bias due to random sequence generation and allocation concealment blinding bias (A1, A3); (2) caregiver blinding and outcome‐assessor blinding (B2, C2); (3) Attrition bias due to incomplete outcome data (D1); and (4) reporting/other bias due to baseline characteristics (A2), random housing (B1), random outcome assessment (C1), selective‐outcome reporting (E1), and any additional concerns, such as funding source or unit‐of‐analysis errors (F1).

This four‐domain scheme follows recent preclinical systematic reviews that collapsed the two blinding items and grouped selective‐reporting and random housing under “other bias” when item‐level reporting was sparse [27, 28, 29, 30]. All ratings were made by the author and double‐checked 1 week later to minimize subjective drift. Overall risk of bias was High if any domain was High, Unclear if at least one domain was Unclear and none was High, and Low only if all four domains were Low.

No statistical publication‐bias analyses were undertaken because meta‐analysis was not conducted. Selective reporting was captured qualitatively within the Reporting/other bias domain.

### 2.7 Data Synthesis & Analytic Approach

Extracted data were synthesized narratively. Each outcome was coded for direction of effect (↑ increase, ↓ decrease, ↔ no difference) according to predefined rules in the finding table. Outcomes reported in ≥ 3 independent studies were designated as sentinel outcomes and tabulated within the four prespecified themes. For cellular composition, Sertoli‐cell “number” was restricted to studies reporting SRY‐box transcription factor 9 (SOX9)‐positive Sertoli cell counts (or an explicit total‐number estimate). The profile‐based nuclei‐per‐tubule metrics were not pooled as Sertoli‐cell number.

Subgroup evidence maps were created for species (mouse, rat) and temperature band (≥ 43°C vs. < 43°C). Each cell displayed *x*/*y*, where *x* was mainly the number of studies reporting a decrease (↓) relative to the control, with the exception of apoptosis findings, where *x* indicated an increase (↑), and *y* was the number of studies that measured the outcome within that subgroup. Because many subgroup strata contained only one or two contributing studies, no comparative conclusions were drawn for strata with *y* < 3; these cells were displayed for transparency only. Cells in which no study measured the outcome were denoted “N/A.” When multiple recovery timepoints were reported, the longest interval ≥ one spermatogenic cycle (35 days mouse; 49 days rat) was used for the primary direction classification.

The longest follow‐up histology description from each study was coded into one of four mutually exclusive categories:

1. *Persistent lesions*: seminiferous‐tubule degeneration or marked germ‐cell loss with no meaningful evidence of recovery;
2. *Partial recovery with residual lesions*: signs of regeneration accompanied by focal defects (e.g., Sertoli‐cell vacuolation, reduced germ‐cell layer);
3. *Near‐normal*: architecture indistinguishable from control or explicitly described as “recovered”;
4. *Indeterminate*: descriptive text insufficient for classification; histology images alone (without explicit author interpretation) were used to infer recovery status.

No quantitative pooling was undertaken because study methods and outcome measures were too heterogeneous for valid meta‐analysis. Instead, the results were summarized in a structured narrative synthesis following the Synthesis Without Meta‐analysis (SWiM) guidance [31].

## 3 Results

The PICO framework guided the inclusion criteria of the evaluated studies. The population of interest in the selected studies was male rodents, particularly mice and rats, that had been subjected to single transient mild local testicular or scrotal hyperthermia. The studies evaluated the impact of thermal stress on the SSC or spermatogenesis recovery endpoint at the time point of interest, extending over one or more spermatogenic cycles. This allowed examination of the reversibility of SSC damage and spermatogenesis recovery following the reviewed heat model.

### 3.1 Study Selection

Database searches retrieved 1320 records; 709 duplicates were removed, leaving 611 unique titles/abstracts for screening (Figure 1). Title‐ and abstract‐level screening excluded 590 records that did not meet the inclusion criteria (Table S2). Twenty‐one articles underwent full‐text assessment, and three were excluded for ineligible heat protocols or outcomes (Table S3). Ultimately, 18 studies met all eligibility criteria and were included in the qualitative synthesis.

### 3.2 Study Characteristics

Eighteen controlled animal studies met the prespecified PICO criteria (Table 1). Most were conducted in Iran (7 studies, 39%) [32, 33, 34, 35, 36, 37, 38], followed by Brazil (3, 17%) [23, 39, 40], China (3, 17%) [41, 42, 43] and Japan (2, 11%) [44, 45], as well as single studies from Spain, Australia, and the United States [11, 46, 47]. All experiments used male rodents, either mice (*n* = 10) or rats (*n* = 8), and a single, local scrotal hyperthermia. Heating temperatures clustered at 43°C (14 studies) or 42°C (4 studies), with exposure duration ranging from 12 to 30 min. Recovery was assessed from 6 h to 110 days, with every study examining at least one timepoint equal to or exceeding a full spermatogenic cycle (35 days in mice; 49 days in rats). Comparators were predominantly nonheated controls, with one study contrasting heat alone versus heat + magnetic field co‐exposure [23]. Reporting frequencies for the prespecified themes were the following: Structural integrity 14/18 (histology reported in 12 studies), Endocrine/apoptosis 8/18 (testosterone and apoptotic tubules each 6), Stereology 5/18 (all Sertoli cell counts), and Sperm parameters 5/18 (motility shown in all). Subsequent sections presented risk‐of‐bias profiles and outcome patterns within these four themes.

### 3.3 Results by Outcome Theme

#### 3.3.1 Structural Integrity

Histological integrity was assessed in 12 studies (8 mouse, 4 rat) (Table 2). Four studies reported persistent seminiferous tubule (ST) degeneration that extended beyond one full spermatogenic cycle; each also recorded significant decreases in ST diameter, testis weight, or index [32, 36, 38, 43]. Four studies described partial recovery with residual lesions [23, 35, 39, 40], while three reported near‐normal architecture [11, 41, 47]. One study provided an indeterminate description of “restoration progress to some level” without presenting micrographs or a detailed textual interpretation [37].

**TABLE 2:** Structural integrity.

Outcome/study	Kheradmand et al. 2011 (Rat, 43°C × 15 min, 60 days FU RoB = Unclear)	Liu et al. 2012 (Mouse, 42°C × 30 min, 42 days FU, RoB = Unclear)	Queiroz et al. 2013 (Mouse, 43°C × 15 min, 60 days FU, RoB = High)	Tenorio et al. 2013 (Mouse, 43°C × 12 min, 60 days FU, RoB = Unclear)	Chihara et al. 2015 (Mouse, 43°C × 20 min, 60 days FU, RoB = High)	Mohajeri and Kaffashi 2015 (Mouse, 43°C × 15 min, 50 days FU, RoB = Unclear)	Dong et al. 2016 (Mouse, 43°C × 20 min, 35 & 70 days FU, RoB = High)	Moradpour et al. 2018 (Rat, 43°C × 15 min, 60 days FU RoB = High)	Aldahhan et al. 2019 (Rat, 43°C × 15 min, 56 & 98 days FU, RoB = High)	Hasani et al. 2020 (Mouse, 43°C × 30 min, 70 days FU, RoB = Unclear)	Khosravi et al. 2020 (Mouse, 43°C × 30 min, 70 days FU, RoB = Unclear)	Lee et al. 2020 (Mouse, 42°C × 15 min, 42 days FU, RoB = High)	Zhang et al. 2020 (Mouse, 42°C × 20 min, 35 days FU, RoB = Unclear)	Melo et al. 2022 (Rat, 42°C × 12 min, 60 days FU, RoB = Unclear)
Histological integrity	Partial recovery	Near‐normal	Partial recovery	Partial recovery	—	Persistent	—	Indeterminate	Near‐normal	Persistent	Persistent	Near‐normal	Persistent	Partial recovery
Seminiferous‐tubule (ST) diameter	↔	—	—	—	—	↓	—	—	—	↓	↓^c^	—	—	—
Testis weight	—	—	↓^b^	↓^a^	↓	—	—	—	↓	↓	↓	—	—	—
Testis index (gonadosomatic index)	—	—	—	—	—	—	↓ 35d & ↔ 70d	—	—	—	—	↓	↓	—
Histology raw findings	Severe ST degeneration	Germinal degeneration & interstitial oedema	Recovery with focal loss	Minor lesions + regeneration	Major lesions + partial recovery	Mostly normal	No detectable difference	Recovery noted—no data						
Histolog code	Persistent	Persistent	Partial recovery	Partial recovery	Partial recovery	Near‐normal	Near‐normal	Indeterminate						

*Note:* ↑ increased/↓ decreased/↔ unchanged/— not reported.

^a^ Testis weight (inferred data).

^b^ Difference not statistically significant (*p* > 0.05) likely due to low sample size.

^c^ Lumen diameter.

Species patterns were divergent, with all four rat studies demonstrating substantial structural recovery by at least 49 days, whereas mouse studies were heterogeneous, with 2/8 reaching near normal morphology, 4/8 retaining severe germinal epithelium degeneration, and 2/8 showing relative recovery with minor or major lesions.

#### 3.3.2 Stereology

Five studies quantified testicular cell populations in mice, whereas no stereological outcomes were reported in rats (Table 3). Three of the five detected significant reductions in SSCs, meiotic/post‐meiotic germ cells, and Leydig cells; in two of these, the deficits persisted for two full spermatogenic cycles, indicating a long‐term impact of hyperthermia. Sertoli cell counts were more variable, remaining unchanged in 3/5 studies and decreased in 2/5. This variability likely reflects the following methodological heterogeneity: numerical density stereology (3/5), SOX9‐positive Sertoli counts (1/5), and a corrected “cells per tubule cross section per unit tubule length” approach (1/5).

**TABLE 3:** Stereology.

Outcome/study	Queiroz et al. 2013 (Mouse, 43°C × 15 min, 60 days FU, RoB = High)	Chihara et al. 2015 (Mouse, 43°C × 20 min, 60 days FU, RoB = High)	Hasani et al. 2020 (Mouse, 43°C × 30 min, 70 days FU, RoB = Unclear)	Ilkhani et al. 2020 (Mouse, 43°C × 30 min, 35 days FU, RoB = Unclear)	Khosravi et al. 2020 (Mouse, 43°C × 30 min, 70 days FU, RoB = Unclear)
Numbers of SSC	—	—	↓↓	↓↓	↓↓
Pachytene spermatocytes (PSyt)	—	—	↓↓	↓↓	↓↓
Round spermatids (rST)	—	—	↓↓	↓↓	↓↓
Leydig cell (LC) number	—	—	↓	↓	↓
Sertoli cells (SC)/SOX9‐positive SCs number	↔	↓	↓	↔	↔
Method of SC counting	The corrected count of SCs per ST cross section, section thickness and the total STs length	Sox9 labeled	Numerical density	Numerical density	Numerical density

*Note:* ↑ increased/↓ decreased/↔ unchanged/— not reported.

#### 3.3.3 Endocrine & Apoptotic Markers

Eight studies evaluated endocrine or apoptotic markers following hyperthermia at 43°C for 12–30 min (Table 4). Six studies quantified serum or plasma testosterone, and six assessed the percentage of apoptotic tubules, with four studies reporting both outcomes. Testosterone fell significantly in three mouse studies at 50 days or more after heat, whereas two mouse and one rat study reported no change at similar follow‐up (60 and 98 days, respectively). An elevated apoptotic tubule percentage was observed in 5/6 experiments, with two mouse studies showing this increase for two full spermatogenic cycles (70 days). Methodological heterogeneity was present, as testosterone was measured by ELISA in four studies (2 decreased, 2 unchanged) and by radioimmunoassay (RIA) in two studies (1 decreased, 1 unchanged). Apoptosis detection methods, including TUNEL (4), Nile Blue (1), and ssDNA fragmentation (1), yielded broadly consistent findings, with the only unchanged result arising from the TUNEL‐based rat study at 98 days. Overall, rats exhibited milder endocrine disruption, but a similar apoptotic response, when compared with mice. The choice of testosterone assay did not appear to influence the outcome direction.

**TABLE 4:** Endocrine & Apoptosis markers.

Outcome/study	Queiroz et al. 2013 (Mouse, 43°C × 15 min, 60 days FU, RoB = High)	Tenorio et al. 2013 (Mouse, 43°C × 12 min, 60 days FU, RoB = Unclear)	Chihara et al. 2015 (Mouse, 43°C × 20 min, 60 days FU, RoB = High)	Mohajeri and Kaffashi 2015 (Mouse, 43°C × 15 min, 50 days FU, RoB = Unclear)	Aldahhan et al. 2019 (Rat, 43°C × 15 min, 56 & 98 days FU, RoB = High)	Hasani et al. 2020 (Mouse, 43°C × 30 min, 70 days FU, RoB = Unclear)	Ilkhani et al. 2020 (Mouse, 43°C × 30 min, 35 days FU, RoB = Unclear)	Khosravi et al. 2020 (Mouse, 43°C × 30 min, 70 days FU, RoB = Unclear)
Serum or plasma testosterone	↔	↔	—	↓	↔	↓	—	↓
% TUNEL/ssDNA‐positive tubules	↑ GCs nuclear fragmentation	—	↑	—	↔	↑	↑	↑
Testosterone assay/Apoptosis detection method	ELISA/Nile's Blue	ELISA/—	—/ssDNA	RIA/—	RIA/TUNEL	ELISA/TUNEL	—/TUNEL	ELISA/TUNEL

*Note:* ↑ increased/↓ decreased/↔ unchanged/— not reported. Testosterone assay per study: ELISA = 4, RIA = 2. Apoptosis detection: TUNEL = 4, Nile Blue = 1, ssDNA = 1.

#### 3.3.4 Sperm Parameters

Five mouse studies investigated sperm metrics (Table 5), but no rat studies met the criteria. All five studies assessed sperm motility. Two reported a decline at 35 days (2/2) after thermal exposure at 43°C for 20–30 min, including one study that also extended the follow‐up to 70 days [42]. At approximately 60 days (*n* = 4), the results diverged, as motility remained unchanged in the computer‐assisted semen analyzer (CASA)‐based study [46] and in one manual‐count study [42], whereas it was still reduced in the other two manual‐count investigations (2/4). At approximately 35 days, sperm count/concentration had declined in all four studies that measured it, while viability dropped in 3/4 and morphology showed distortions in 2/3. DNA integrity showed a similar pattern of impairment, as the percentage of apoptotic sperm was elevated in 2/3 studies (both Sperm Chromatin Dispersion [SCD]‐based) and was unchanged in one TUNEL assay at 60 days. Methodological heterogeneity was moderate, as one study used a CASA + Bürker hemocytometer, whereas the other four employed manual counting chamber protocols, with two using eosin–nigrosine viability staining. Nevertheless, the direction of the effect was broadly consistent across all methods.

**TABLE 5:** Sperm parameters.

Outcome/study	Pérez‐Crespo et al. 2008 (Mouse, 42°C × 30 min, 60 days FU, RoB = Unclear)	Dong et al. 2016 (Mouse, 43°C × 20 min, 35 & 70 days FU, RoB = High)	Hasani et al. 2020 (Mouse, 43°C × 30 min, 70 days FU, RoB = Unclear)	Ilkhani et al. 2020 (Mouse, 43°C × 30 min, 35 days FU, RoB = Unclear)	Khosravi et al. 2020 (Mouse, 43°C × 30 min, 70 days FU, RoB = Unclear)
Sperm count/concentration ·	↓	—	↓	↓	↓
Motility (total & progressive)	↔	↓ 35 days & ↔ 70 days	↓	↓	↓
Morphology	—	↔ 70 days	↓	↓	—
Viability	↔	—	↓	↓	↓
% Apoptotic sperm (SCD index/TUNEL‐positive)	↔	—	↑	—	↑

*Note:* ↑ increased/↓ decreased/↔ unchanged/— not reported.

### 3.4 Risk of Bias Within Studies

Figure S1 shows the distribution of Low, Unclear and High judgments; individual ratings are in Table S4 and primary reasons in Table S5. Sequence/allocation (RoB_seq) was Unclear in all 18 studies, as none described random sequence generation or allocation concealment. Blinding (RoB_blind) was likewise Unclear in 18/18, as caregiver blinding and outcome assessor masking were never fully reported (statements such as “histology scored blind” did not meet Low criteria). Incomplete outcome data (RoB_inc) were mostly Low (13/18, 72%), reflecting complete sample size reporting and minimal attrition. All papers provided baseline animal characteristics. Reporting/other bias (RoB_rep/oth) had the poorest profile, with 7/18 (39%) High, 10/18 (56%) Unclear, and 1/18 (6%) Low. The High ratings stemmed from selective outcome reporting, unit of analysis errors, or genotype/co‐intervention confounders. The Unclear ratings mainly reflected the absence of random housing information. Applying the pre‐specified algorithm (any High → High overall), 7/18 studies were High overall risk and 11/18 were Unclear; no study achieved a Low overall risk.

### 3.5 Subgroup Patterns (Species and Temperature)

#### 3.5.1 Structural Integrity Subgroup Analysis

The structural integrity subgroup patterns are illustrated in Table 6. Heat‐induced damage was more frequent in mice than in rats, as histology deteriorated in 6/8 mouse comparisons versus 2/4 rat. Every mouse study that measured ST diameter (3/3) or testis weight (6/6) found reductions, whereas the single rat measurement of ST diameter showed no change and one rat study reported reduced testis weight. Temperature appeared to amplify the effect, with 43°C exposure in both species (mouse & rat) resulting in decreases in almost every theme outcome (histology 6/8; ST diameter 3/4; testis weight 6/6; testis index 1/1). However, the thermal impact was mild at 42°C, with disrupted histology observed in 2/4 reports and a decline in the testis index in 2/2, while ST diameter and testis weight data were not reported at this temperature. Overall, these results indicate a clear species gradient (mouse > rat) and a temperature threshold, with measurable structural damage markedly more likely at 43°C, particularly for tubular diameter and testis weight.

**TABLE 6:** Ratio of studies reporting a significant decrease (↓) to studies measuring each structural integrity outcome within key subgroups.

Outcome ↓	Mouse ↓	Rat ↓	43°C ↓	42°C ↓
Histology integrity	6/8	2/4	6/8	2/4
ST diameter	3/3	0/1	3/4	NR
Testis weight	5/5	1/1	6/6	NR
Testis index	3/3	NR	1/1	2/2

*Note:* Fractions are calculated as (studies showing ↓)/(studies that reported the outcome within that subgroup). Studies that did not measure the outcome are not included in the denominator. NR = this outcome not reported in that subgroup.

#### 3.5.2 Stereology Subgroup Analysis

All stereological outcomes were reported only in the murine model at 43°C; no rat data were recorded at either temperature (Table 7). The number of SSCs, pachytene spermatocytes, round spermatids, and Leydig cells decreased in all three studies (3/3). Sertoli cell quantification was less consistent, showing decreases in 2/4 assessments and remaining unchanged in the remainder. No stereological data were reported at 42°C in mice. Collectively, these findings highlight that a single transient 43°C heat treatment can produce long‐term depletion across most testicular cell populations in mice.

**TABLE 7:** Ratio of studies reporting a significant decrease to studies measuring each stereology outcome within key subgroups.

Outcome ↓	Mouse ↓	Rat ↓	43°C ↓	42°C ↓
Numbers of SSC	3/3	NR	3/3	NR
Numbers of Psyt	3/3	NR	3/3	NR
Numbers of rST	3/3	NR	3/3	NR
SC/SOX9‐positive number	2/4	NR	2/4	NR
LC number	3/3	NR	3/3	NR

*Note:* Fractions are calculated as (studies showing ↓)/(studies that reported the outcome within that subgroup). Studies that did not measure the outcome are not included in the denominator. NR = this outcome not reported in that subgroup.

#### 3.5.3 Endocrine & Apoptosis Subgroup Analysis

The endocrine & apoptosis subgroup patterns are shown in Table 8. Testosterone declined in 3/5 mouse studies, whereas the single rat study recorded no change. The percentage of apoptotic tubules increased in all five mouse reports but remained unchanged in the lone rat experiment. Temperature analysis supported the following threshold effect: at 43°C, apoptosis increased in 5/6 comparisons, whereas no apoptosis data were available at 42°C, precluding comparison. Overall, endocrine suppression and tubular apoptosis were observed primarily in mice and at the higher temperature (43°C).

**TABLE 8:** Ratio of studies reporting a significant decrease to studies measuring each endocrine and apoptosis outcome within key subgroups.

Outcome ↓	Mouse ↓	Rat ↓	43°C ↓	42°C ↓
Serum or plasma testosterone	3/5	0/1	3/6	NR
% TUNEL/ssDNA‐positive tubules	↑5/5	0/1	↑5/6	NR

*Note:* Fractions are calculated as (studies showing ↓)/(studies that reported the outcome within that subgroup). Studies that did not measure the outcome are not included in the denominator. NR = this outcome not reported in that subgroup. 0 = no change.

#### 3.5.4 Sperm Parameter Subgroup Analysis

Table 9 summarizes the patterns of sperm parameters by subgroup. All five studies evaluated murine sperm metrics, whereas no rat data were reported. In detail, sperm count/concentration diminished in every mouse comparison (4/4) at both 42°C and 43°C. However, the other functional indices revealed the following temperature‐dependent impairments: at 43°C, motility declined in 4/5 studies, morphology in 2/3, and viability in 3/4, whereas none of these parameters decreased at 42°C. Sperm DNA integrity showed a similar pattern, with apoptotic‐sperm percentages showing elevations in 2/2 experiments at 43°C but no changes in the single 42°C experiment. Together, these analyses indicate that the adverse impact of hyperthermia on sperm quality intensifies at 43°C and affects multiple functional parameters beyond the universal reduction in sperm count.

**TABLE 9:** Ratio of studies reporting a significant decrease (↓) to studies measuring each sperm outcome within key subgroups.

Outcome ↓	Mouse ↓	Rat ↓	43°C ↓	42°C ↓
Sperm count or concentration	4/4	NR	3/3	1/1
Motility (total & progressive)	4/5	NR	4/4	0/1
Morphology	2/3	NR	2/3	NR
Viability	3/4	NR	3/3	0/1
% Apoptotic sperm (SCD index/TUNEL‐positive)	↑2/3	NR	↑2/2	↑0/1

*Note:* Fractions are calculated as (studies showing ↓)/(studies that reported the outcome within that subgroup). Studies that did not measure the outcome are not included in the denominator. NR = this outcome not reported in that subgroup. Cells with denominator < 3 indicate sparse evidence; no comparative subgroup inference is made.

### 3.6 Summary of Evidence

Across 18 rodent studies, a single exposure of local scrotal hyperthermia produced a coherent but temperature‐ and species‐dependent pattern of injury.

#### 3.6.1 Structural Integrity

Histology, seminiferous‐tubule diameter and testis weight most often worsened in mice at 43°C (histology ↓ 6/8; ST diameter ↓ 3/3; weight ↓ 6/6), whereas these parameters were less affected in rats (histology ↓ 2/4; weight ↓ 1/1; ST‐diameter 0/1 unchanged). Only 4/12 histology comparisons showed near‐normal architecture at the longest follow‐up; the remainder (8/12) still exhibited persistent or partially recovered lesions.

#### 3.6.2 Stereology

All mouse studies that quantified cellular composition at 43°C reported long‐term depletion of SSCs, meiotic/post‐meiotic germ cells, and Leydig cells (each ↓ 3/3). The Sertoli‐cell loss was partial (↓ 2/3). No stereology data were available at 42°C or for rats.

#### 3.6.3 Endocrine & Apoptosis

Testosterone responses were heterogeneous, showing decreases in 3/5 mouse studies but no changes in the single rat study, whereas tubular apoptosis increased in 5/6 mouse experiments at 43°C but remained unchanged in the lone rat experiment.

#### 3.6.4 Sperm Parameters

Sperm count or concentration declined in all four mouse studies that measured it, at both 42°C and 43°C. Functional quality metrics deteriorated chiefly at 43°C, with motility ↓ 4/5, viability ↓ 3/4, morphology ↓ 2/3, and apoptotic sperm ↑ 2/2, None of these parameters declined at 42°C.

#### 3.6.5 Integrative View

Structural repair was not consistently concordant with functional recovery. Despite the relative restoration of ST morphology (a minority, 4/12 comparisons), impairment persisted in the functional endpoints, including sperm motility, count, or DNA integrity. Conversely, most studies still showed histological damage (8/12), reinforcing the notions that structural and functional healing frequently progress contemporaneously and that functional deficits can persist despite apparently repaired tissue. Taken together, these results reveal a clear species gradient (mouse > rat) and a temperature threshold, with measurable structural and functional damage markedly more likely at 43°C. These themes frame the mechanistic and translational implications explored in Section 4.

## 4 Discussion

### 4.1 Principal Findings

This systematic review scrutinized experimental rodent studies that investigated the effect of a single episode of acute mild scrotal hyperthermia on SSC‐driven recovery across one or more spermatogenic cycles. Although a minority of the comparisons at the longest follow‐up report that the SCCs show near‐normal histological architecture, this structural normalization is not consistently accompanied by functional recovery, indicating that structural repair alone does not necessarily reflect the restoration of spermatogenic output. In parallel with this structure–function discordance, the most quantitative cellular evidence, restricted to mouse stereology at 43°C, indicates a long‐term loss of many different germ cells, including SSCs, meiotic/post‐meiotic cells, and Leydig cells, but only a partial loss of Sertoli cells. Endocrine responses are heterogeneous, whereas tubular apoptosis increases more consistently in mouse experiments at 43°C while remaining unchanged in the single rat experiment. Collectively, these findings align with a model in which SSCs are not uniformly eliminated by acute heat; rather, they undergo transient perturbations and/or functional impairments that shape the trajectory of recovery. However, confidence in these inferences is constrained by the substantial risk of bias and heterogeneity in outcome ascertainment, follow‐up windows, and SSC‐specific endpoints.

### 4.2 Structural Integrity and Recovery Interpretation

A key interpretive point revealed by this review is the inconsistency in recovery signals. Notably, while structural endpoints often suggest partial restoration, this evidence is frequently incomplete and is more consistently observed in mice than in rats. Other histological markers, despite showing heat‐induced changes, are insufficient as standalone indicators of SSC‐driven recovery across the available literature. This ambiguity is often compounded by reporting gaps, such as missing micrographs, or by sparse textual interpretations that hinder cross‐study comparisons and fall short of current transparency standards for in vivo research [48]. Ultimately, because functional impairments often persist even when the structure appears to improve, SSC‐driven restoration may be overestimated when relying on morphological endpoints alone. These findings reinforce the need for standardized reporting protocols and greater precision in quantitative cellular metrics.

### 4.3 Quantitative Cellular Recovery (Stereology)

Quantitative cellular data provide a more direct basis for interpreting whether apparent architectural repair reflects genuine SSC‐driven recovery. This is particularly evident in mouse stereological studies conducted at 43°C, which document long‐term depletion of germ‐cell populations, including SSC‐labeled populations. Where histology is reported in these investigations, it also describes severe seminiferous‐tubule degeneration, supporting concordance between overt architectural injury and measurable cellular loss under the most injurious dosing conditions. However, because stereology is generally not performed in studies where histology approaches near‐normal architecture at the longest follow‐up, the extent to which structural normalization truly reflects the restoration of cellular composition remains unclear. Consequently, apparent recovery based on morphology alone may mask persistent cellular deficits; thus, this possibility cannot be resolved without quantitative cellular assessment in testes that appear histologically recovered.

These discrepant impressions of SSC “resistance” versus “vulnerability,” rather than representing a simple binary state, appear to reflect differences in key methodological aspects, such as thermal dosing, follow‐up alignment and outcome definitions. Early studies with short post‐heat observation windows report little apparent change in basal spermatogonial compartments (as defined histologically at the time), thereby contributing to the longstanding impression of relative SSC resistance [9, 10]. Nonetheless, preserved counts at early timepoints do not preclude acute functional perturbation. In the context of the present synthesis, in which functional endpoints often lag behind and where the most quantitative in vivo data at 43°C indicate longer‐term depletion of multiple germ‐cell populations, a key interpretive possibility is that early non‐apoptotic SSC perturbations can precede and shape later incomplete recovery. In vitro evidence indicates that, within hours after short exposures, brief hyperthermia can rapidly disrupt SSC transcriptional and cell‐cycle programmes, including JAK–STAT–linked changes and transient S‐phase accumulation, without invoking an immediate apoptotic signature [17]. Viewed in this way, these early perturbations provide a coherent bridge between apparently preserved early SSC numbers and later impaired recovery trajectories. Thus, the “SSC resistance versus vulnerability” divergence may represent endpoint selection and timing, with “resistance” sometimes reflecting early quantitative preservation rather than preserved SSC competence.

### 4.4 Endocrine, Apoptotic and Sperm Functional Outcomes

Although apoptosis and endocrine outcomes were summarized together in the Results for conciseness, apoptosis is discussed here together with cellular composition because it can mechanistically correlate with germ‐cell depletion and incomplete recovery. Increased tubular cell death is detected predominantly within the seminiferous epithelium and, where micrographs are available (4/5 studies), it is consistent with an involvement of heat‐susceptible meiotic and post‐meiotic populations (pachytene spermatocytes and round spermatids). This has been inferred from cellular morphology and location and is in keeping with the extensive body of literature [8]. This pattern also coincided with reduced counts of these cells (Tables 3 and 4) and with the broadly consistent apoptotic response across studies, despite differences in their detection methods. This may also be consistent with a heat‐induced germ cell quality‐control response, in which damaged or abnormal germ cells are selectively eliminated following thermal stress. In this context, heat shock factor‐1 (HSF1) has been implicated in thermally induced apoptotic quality control in male germ cells, particularly in pachytene spermatocytes [49]. In these apoptosis studies, the significant tubular apoptotic signal persists for up to two spermatogenic cycles, supporting the possibility that regenerative recovery may be incomplete and could reflect SSC‐level and/or niche‐level dysfunctions rather than a purely transient loss of heat‐susceptible meiotic/post‐meiotic cells.

Endocrine and sperm endpoints further illustrate variable functional recovery in response to heat stress. Testosterone responses are heterogeneous and do not show consistent concordance across species or follow‐up periods, despite the evidence of Leydig cell vulnerability in the quantitative cellular data. By contrast, sperm output provides the clearest functional signal, as sperm count/concentration is consistently reduced where assessed. Furthermore, at 43°C, additional quality metrics (motility, viability, morphology, and DNA integrity) are more frequently disrupted, supporting a temperature threshold for a durable functional compromise beyond any early histological change. Only two studies with a 70‐day follow‐up have measured both testosterone and sperm metrics, and they report concurrent testosterone reduction and sperm impairment, consistent with the salient role of testosterone in supporting spermatogenesis. The persistence of testosterone and sperm abnormalities beyond one spermatogenic cycle may indicate a longer‐term thermal impact that could, at least in part, reflect SSC‐level and/or niche‐level disturbances, even when histology suggests relatively good architectural recovery.

### 4.5 Species and Temperature Subgroup Patterns

The studies evaluated in this review suggest that the mouse–rat gradient and the apparent 43°C “threshold” are likely to reflect a combination of true biological susceptibility and methodological dispersion, rather than representing a simple binary heat–no heat effect. At the upper end of scrotal heating, even a 1°C increment can be consequential because spermatogenesis is normally supported below the core body temperature through dedicated thermoregulatory mechanisms (including local counter‐current transfer), with modest upward shifts capable of causing a disproportionate destabilization of germ cell homeostasis [50, 51]. Species‐specific recovery dynamics may also contribute to the more persistent abnormalities observed in mice because the shorter spermatogenic duration in mice than in rats can compress injury–repair trajectories and complicate cross‐species alignment of follow‐up windows to equivalent biological stages [52]. In addition, heterogeneity in thermal dosing, core‐temperature control and outcome ascertainment, together with the scarcity of SSC‐resolved endpoints, may inflate apparent between‐study gradients. Accordingly, while the aggregate pattern remains consistent with more reliable detectability of injury at 43°C and more persistent effects in mice than in rats, these gradients should be interpreted cautiously as reflecting an interplay between biological vulnerability and experimental design [8, 48].

### 4.6 Risk of Bias and Methodological Constraints

As added complications, methodological heterogeneity, together with risk of bias, materially tempers how much confidence can be placed in the apparent mouse–rat gradient and the 43°C signal [53]. The use of an animal study–specific framework (SYRCLE) confirms that key domains that are frequently unclear or that present a high risk of bias most commonly involve random sequence generation and allocation concealment, blinded outcome assessment (particularly for semi‐quantitative histology), incomplete outcome data/attrition handling, and selective outcome reporting, thereby reflecting limitations in both design safeguards and reporting transparency [54]. This matters because animal studies that do not utilize randomization and blinding are more likely to report between‐group differences, particularly where outcomes are susceptible to observer influence [55, 56]. Moreover, incomplete reporting of bias‐reduction methods has been associated with inflated effect sizes in animal research, further complicating any inferences when endpoints are variably ascertained [57]. These factors necessitate methodological standardization in future research, including prespecified analysis plans and explicit reporting of randomization, blinding, and subject attrition. Standardized documentation of thermal dosing and core temperature control is also needed to improve comparability and interpretability across studies. This approach would be consistent with widely endorsed minimum standards for improving the interpretability and predictive value of preclinical research [58, 59].

### 4.7 Integrated Interpretation, Strengths and Limitations

Across the included rodent experiments, the discordance between apparent structural repair and persistent endocrine and sperm abnormalities suggests that architectural improvement alone is an incomplete surrogate for spermatogenic recovery. This structure–function gap is most plausibly explained by residual cellular and molecular injury that is not captured by gross histological normalization. This could include incomplete repopulation of key germ cell compartments and/or persistent dysfunction within supporting somatic or niche elements that govern SSC fate. Consistent with this interpretation, the most sustained functional deterioration clusters at 43°C, where sperm quality and/or output frequently remain impaired beyond one spermatogenic cycle. In studies that have reported paired endocrine–sperm datasets at long follow‐up, the reduced testosterone coincides with impaired sperm endpoints, supporting an enduring disturbance rather than a transient maturational delay.

Mechanistically, acute heat can activate coordinated stress‐response programmes within the seminiferous epithelium to promote germ‐cell loss and reduce downstream sperm output, even when tubule architecture later appears partially restored [60]. In parallel, early post‐heat transcriptional and post‐transcriptional reprogramming (including in vitro alterations in miRNA profiles after transient hyperthermia and disruption of SSC proliferation/differentiation) provides a credible route by which a brief insult can be “stored” biologically and expressed later as altered regeneration kinetics and persistent sperm defects [16, 61, 62]. Heat‐induced apoptotic and proliferative perturbations have also been demonstrated in spermatogenic cell‐line models, supporting the broader plausibility of germ‐cell stress responses under hyperthermic challenge, although not necessarily SSC‐specific ones [63, 64]. Moreover, evidence that the male meiotic prophase depends on specialized networks driven by heat‐shock factors, even under non‐stress conditions, supports the concept that meiotic progression is governed by tightly regulated stress‐related transcriptional machinery [65]. Thus, the persistence of testosterone and sperm abnormalities beyond one spermatogenic cycle is consistent with SSC‐level and/or niche‐level disturbances. Because these are not reliably reflected by histology, this reinforces the need to define “recovery” using functional and quantitative cellular endpoints rather than architecture alone.

To the best of the author's knowledge, and based on database searches up to submission, this systematic review is the first to restrict article inclusion strictly to those involving in vivo experimental rodent studies. In particular, studies are included that extend beyond the direct application of acute local scrotal hyperthermia and the assessment of SSC‐relevant or spermatogenesis‐relevant recovery across at least one full spermatogenic cycle using a PICO‐driven eligibility framework. The principal strength of this review lies in its deliberate cycle‐aligned focus, which provides a biologically meaningful window for distinguishing transient germ cell loss from durable impairment of SSC‐driven regeneration. Furthermore, the theme‐based synthesis enables structured integration of structural, cellular, endocrine/apoptotic, and sperm functional endpoints across heterogeneous outcome types.

However, several limitations constrain the drawing of specific inferences. These include heat exposure protocols and follow‐up windows that vary substantially, thermal dosing that is often insufficiently standardized or reported, and outcome assessments that frequently rely on semi‐quantitative histology. Quantitative cellular data are also sparse and largely confined to mouse studies at 43°C, whereas SSC‐resolved functional assays are uncommon, and most studies infer SSC status indirectly from morphology or marker expression. Finally, the substantial risk of bias and incomplete reporting across key methodological domains restrict certainty and preclude strong causal claims regarding reversibility, thereby reinforcing the caution that the observed gradients should be interpreted as patterns within an imperfect evidence base rather than definitive effect estimates.

### 4.8 Translational Implications and Future Directions

The rodent evidence has restrained but clinically relevant implications for settings in which human testicular temperature becomes transiently elevated (e.g., febrile illness, sauna/hot bathing, occupational “wet heat” and disorders of scrotal thermoregulation such as varicocele) [8, 66]. Human studies indicate that febrile episodes, sauna exposure, experimental mild testicular heating and chronic wet‐heat exposure can all be followed by delayed, but potentially reversible, deterioration in semen parameters, with recovery trajectories extending over weeks to months [67, 68, 69, 70, 71]. Within this broader context, two translational points emerge: (1) the functional endpoints can remain impaired even when structural recovery is reported; and (2) heat susceptibility is best viewed as graded, with risk shaped by the intensity, duration, and repetition of heat exposure and by baseline thermoregulatory competence [7, 8].

Future work requires explicit structure to address the limitations and recurrent unclear/high‐risk domains identified in the included evidence base. At a minimum, studies should implement and clearly report randomization, allocation concealment (where feasible), blinded outcome assessment, prespecified primary endpoints, sample‐size justification and transparent handling of exclusions and missing data because the pervasive absence or non‐reporting of these safeguards undermines confidence in causal inference regarding in vivo “reversibility” [48, 53, 54, 58]. Thermal dosing should also be standardized and documented with sufficient granularity to permit replication and meaningful between‐study comparisons. This should include explicit reporting of how local scrotal heating is verified and how systemic temperature confounding is controlled. Designs should then test dose responses a priori, ideally with parallel 42°C and 43°C arms and matched exposure durations, rather than making post hoc inferences regarding a “threshold.” Finally, outcome ascertainment should progress beyond semi‐quantitative histology and aim at quantitative cellular readouts of SSC and niche compartments (e.g., stereology and/or validated SSC marker panels, such as ID4/PLZF, with defined counting rules and sampling frames). Where feasible, this should be coupled with SSC‐resolved functional assays, as well as harmonized sperm and endocrine endpoints collected at prespecified biologically meaningful checkpoints (early, one cycle and ≥ 2 cycles) to distinguish transient perturbation from durable impairment of SSC‐driven regeneration.

## 5 Conclusion

The reviewed rodent investigations suggest a graded, temperature‐dependent and species‐related injury pattern following a single episode of local scrotal hyperthermia. In particular, persistent structural and functional impairment is most evident at 43°C and the phenotype is generally milder in rats than in mice. Apparent architectural repair is not reliably concordant with functional recovery, as sperm parameters often remain abnormal even when histology appears near‐normal. Accordingly, the available evidence supports only cautious conclusions regarding reversibility, with recovery frequently being incomplete, endpoint‐specific, and more consistently impaired at 43°C. These findings highlight the need for longitudinal, SSC/niche‐resolved in vivo designs that directly adjudicate stem‐cell competence and recovery across at least one full spermatogenic cycle.

## Conflicts of Interest

The author declares no conflicts of interest.

## Supporting information


**Figure S1:** Risk of bias across SYRCLE domains (*n* = 18).

**Table S1:** Full database search strategies & run dates.

**Table S2:** Primary reasons for excluding 590 records during title/abstract screening.

**Table S3:** Reasons for excluding reports after full‐text eligibility assessment.

**Table S4:** Risk of bias per study.

**Table S5:** Key reasons underpinning risk‐of‐bias judgments across the four consolidated domains (*n* = 18 studies).

## Data Availability

The data that support the findings of this study are available from the corresponding author upon reasonable request.
