# Supplementary figures and images for: Reversibility of Spermatogonial Stem Cell Injury After Single Acute Scrotal Hyperthermia in Mouse and Rat Models: A Systematic Review
Source: Reprod Med Biol. 2026 May 11;25(1):e70055. doi: 10.1002/rmb2.70055 (PMC13160925; doi:10.1002/rmb2.70055)

Supplementary Figure 1

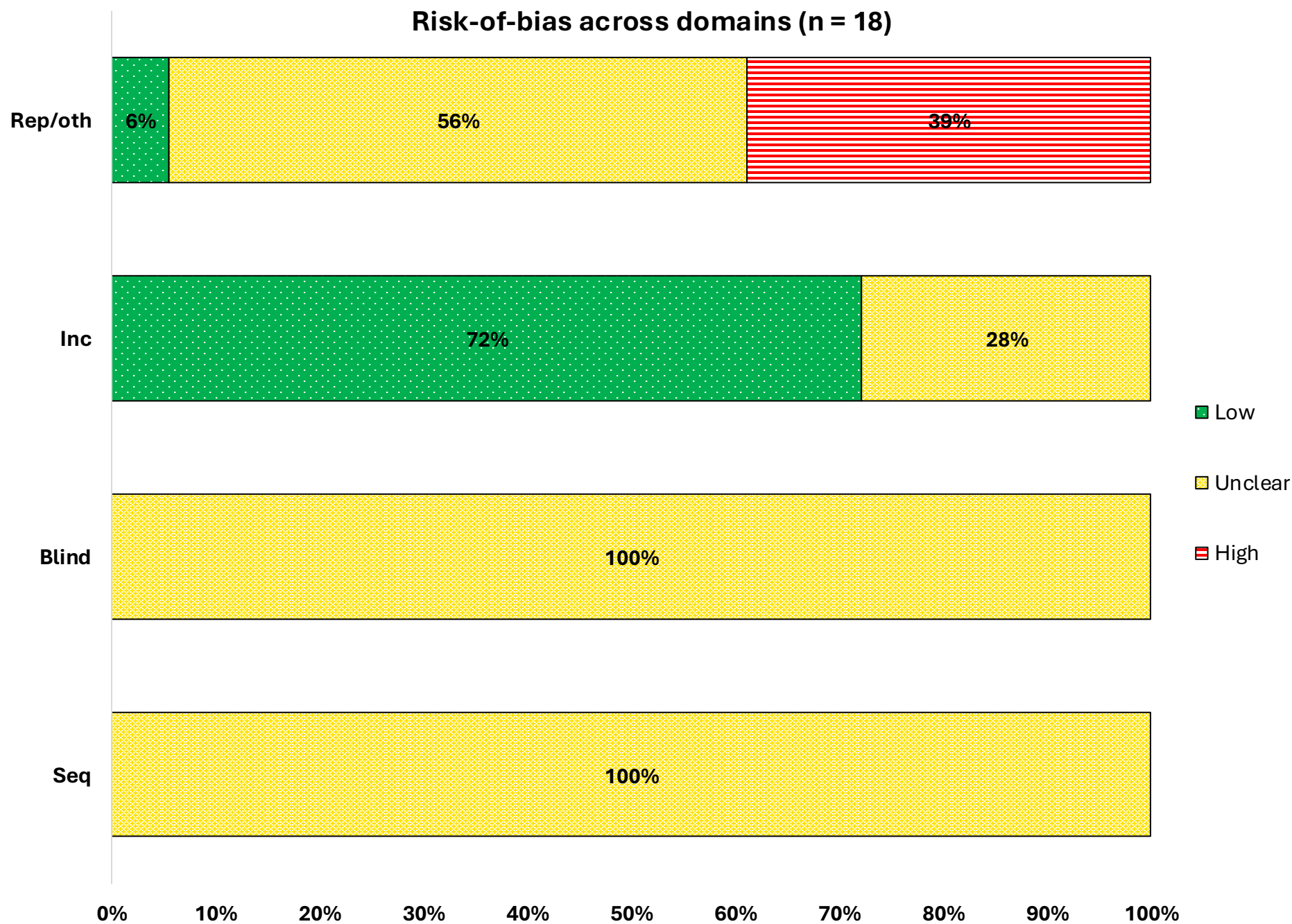

Supplement: Supplementary file 1 — Figure S1: Risk of bias across SYRCLE domains (n = 18). [file RMB2-25-e70055-s006.pdf]
